# Sampling Sub-Diffraction Temperature Gradients with Spectrally Orthogonal Nanoparticle Luminescence

**DOI:** 10.1021/acsphotonics.5c02017

**Published:** 2025-10-23

**Authors:** Benjamin Harrington, Qiwen Xiao, Junyi Lin, Ashley Johnson, Andrea D. Pickel

**Affiliations:** 1 Materials Science Program, 6927 University of Rochester, Rochester, New York 14627, United States; 2 Department of Mechanical Engineering, 6927 University of Rochester, Rochester, New York 14627, United States; 3 The Institute of Optics, 6927 University of Rochester, Rochester, New York 14627, United States; 4 Department of Physics, Texas State University, San Marcos, Texas 78666, United States

**Keywords:** nanothermometry, luminescence thermometry, upconverting nanoparticles, luminescence spectroscopy, thermal metrology

## Abstract

Recording the temperature-dependent luminescence emitted by an isolated single nanoparticle offers one strategy for performing far-field optical thermometry with spatial resolution below the diffraction limit. However, such measurements are inherently restricted to probing the temperature at a single spatial point. Here, we demonstrate an approach to sampling temperature gradients at multiple points within a subdiffraction region by simultaneously collecting the emission from different nanoparticle species with spectrally orthogonal temperature-dependent luminescence. Taking advantage of the narrow spectral bands and wavelength tunability of lanthanide-doped upconverting nanoparticle (UCNP) emission, we use a single laser to excite both NaYF_4_:Yb^3+^,Er^3+^ and NaYF_4_:Yb^3+^,Tm^3+^ UCNPs and concurrently acquire their spectrally distinct temperature-dependent luminescence. The emission spectra and temperature response obtained from tandem UCNP pairs consisting of one NaYF_4_:Yb^3+^,Er^3+^ and one NaYF_4_:Yb^3+^,Tm^3+^ UCNP are in excellent agreement with corresponding measurements using isolated individual UCNPs of each composition. To demonstrate the utility of this approach, we use a tandem pair of UCNPs located ∼108 nm from each other to probe the sharp temperature gradient resulting from laser heating of an isolated silver nanodisk. While the diffraction-limited emission spots of the UCNPs overlap nearly completely, we can distinguish a temperature difference of ∼19 K between their two locations. This capability is particularly applicable to scenarios that would benefit from multiple temperature data points, but where the majority of the sample surface must remain accessible for other purposes, such as in the case of plasmonic and photothermal catalysis.

## Graphical abstract

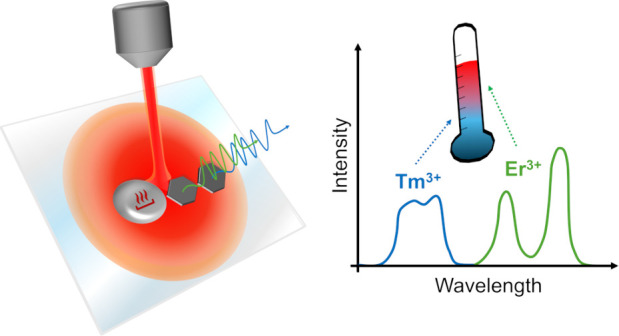

## Introduction

Optical thermometry methods, such as infrared thermography, thermoreflectance, and Raman- or luminescence-based techniques,^1–7^ offer the benefit of collecting the temperature-dependent signal remotely from the far field. However, the diffraction limited spatial resolution of these optical methods is a limiting factor for probing hot spots or steep temperature gradients across wide-ranging applications involving nanoscale features, including microelectronics,^8^ memory devices,^9^ plasmonics,^10^ and intracellular processes.^11^ While we recently demonstrated optical super-resolution nanothermometry based on stimulated emission depletion (STED) imaging,^12^ this method involves wavefront shaping and combining multiple laser beams, increasing the measurement complexity. Additionally, this approach requires coating the sample surface with a layer of luminescent probes, which may be undesirable in situations where access to the sample surface must be preserved. An alternative strategy for performing far-field optical thermometry with spatial resolution below the diffraction limit is to place an isolated single nanoparticle precisely at the location of interest and record its temperature-dependent luminescence.^13–17^ This method works well when knowledge of the temperature at a single critical point is sufficient, but in other cases a single-point measurement is inadequate. For a subset of those cases, the ability to simultaneously probe multiple discrete points within a subdiffraction region would provide temperature heterogeneity information whose value substantially exceeds that of a single-point measurement, with minimal trade-offs in terms of measurement complexity or sample surface obstruction.

One way to achieve this goal is to employ multiple luminescent probes whose temperature-dependent optical responses fall into distinct wavelength ranges and can thus be separated in the spectral domain. Distinguishing individual molecules via spectral separation of their absorption line widths was in fact an early proposed approach to optical super-resolution imaging,^18^ although separation in the time domain ultimately proved more practical, leading to single-molecule localization methods.^19^ Despite the prevalence of time-domain separation for super-resolution imaging, the principle of distinguishing probes via spectral separation has proven useful in other applications, particularly those involving lower labeling densities or with less stringent demands on measurement throughput. One example is nanocomposite heterostructures containing both upconverting nanoparticles (UCNPs) and quantum dots that emit temperature-dependent luminescence in different wavelength ranges, which enabled temperature measurements at two spatially separated locations within a single 110 nm nanocomposite.^20^ However, the ability to sample subdiffraction temperature gradients using multiple nanoparticles placed at distinct locations on a surface has yet to be demonstrated.

Here, we show that multiple UCNPs of different compositions located within a subdiffraction region can be simultaneously excited and their individual temperature-dependent responses can be isolated via spectral separation. Like single-particle measurements, this method requires only a single Gaussian excitation beam and thus involves much less complex instrumentation than STED, while still providing multipoint temperature information that single-particle measurements fundamentally cannot access. We focus specifically on tandem pairs consisting of one NaYF_4_:Yb^3+^,Er^3+^ UCNP and one NaYF_4_:Yb^3+^,Tm^3+^ UCNP, as illustrated in Figure 1a, but in principle this approach can be extended to other UCNP compositions and expanded to incorporate additional spectrally orthogonal temperature-dependent luminescence signals. UCNPs are commonly codoped with multiple lanthanide ion species. Frequently, one dopant (the sensitizer) serves as the absorber and the other (the activator) serves as the emitter. Separating the roles of absorber and emitter facilitates the selection of multiple UCNP compositions that absorb at the same wavelength, enabling the use of a single excitation laser, but emit at distinct wavelengths. UCNP excitation and emission wavelengths can be tuned by combining different sensitizers and activators from the library of available lanthanide ion dopants or by varying the dopant concentrations.^21^ UCNPs also display spectrally narrow emission peaks, minimizing undesirable overlap.^22,23^ We excite both of our Yb^3+^-sensitized UCNP compositions with the same 976 nm laser, while our selected temperature-dependent emission signals occur at green (∼520–550 nm) and blue (∼470–480 nm) wavelengths for Er^3+^ and Tm^3+^ activators, respectively. We systematically validate key aspects of our approach, confirming the absence of detrimental spectral crosstalk even at the high excitation intensities required for single-particle measurements and verifying that the temperature-dependent emission signals for both UCNP compositions are the same whether acquired from individual UCNPs or tandem pairs. We further demonstrate the ability to access subdiffraction temperature information by sampling a steep laser-induced temperature gradient at two different points. We envision that this capability will be especially beneficial in scenarios that involve optical heating or other optical metrology and require maintaining access to the sample surface. Quantifying localized heating during plasmonic photocatalysis while preserving surface access for reacting molecules serves as one motivating example,^24^ but other areas including microelectronics and photonics could similarly benefit from this technique.

**Figure 1:**
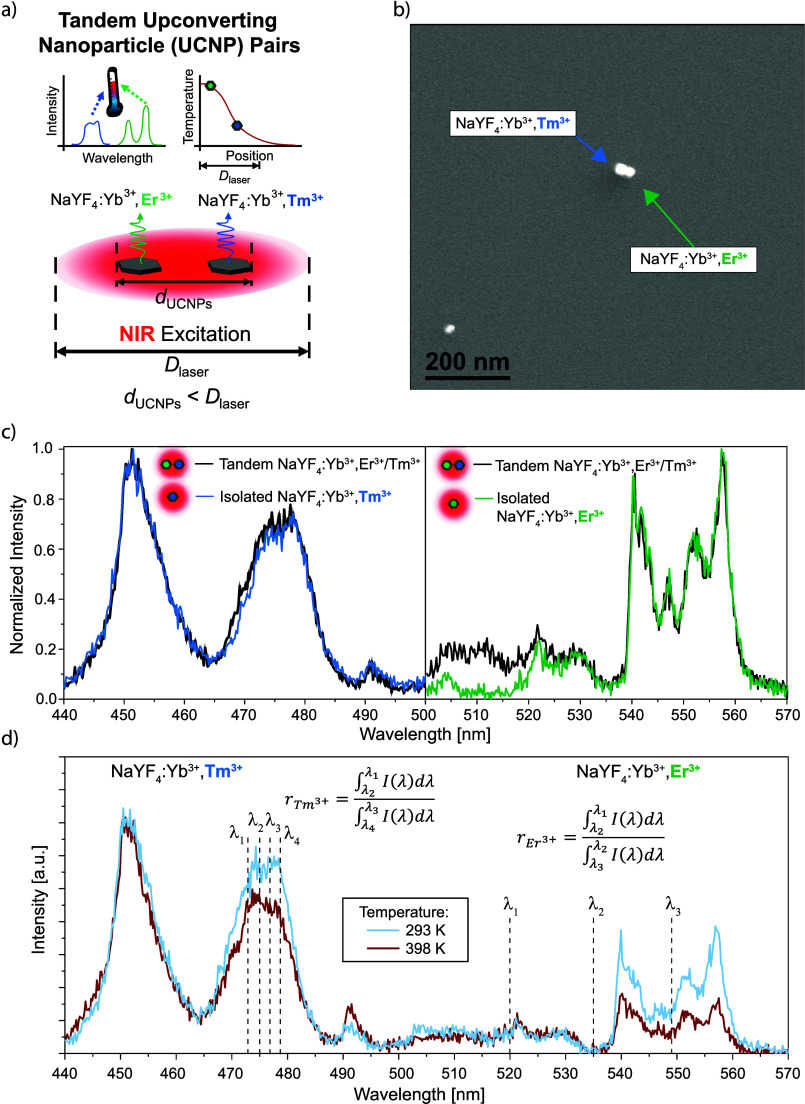
(a) Schematic illustration of a tandem UCNP pair consisting of one NaYF_4_:Yb^3+^,Er^3+^ and one NaYF_4_:Yb^3+^,Tm^3+^ UCNP whose spectrally orthogonal luminescence can be used to sample subdiffraction temperature gradients. The distance between the two UCNPs (*d*_UCNPs_) is smaller than the laser diameter (*D*_laser_). (b) Scanning electron microscope (SEM) image of a tandem UCNP pair. (c) Room temperature emission spectra acquired from the same tandem UCNP pair shown in panel (b) along with emission spectra acquired from isolated individual NaYF_4_:Yb^3+^,Er^3+^ and NaYF_4_:Yb^3+^,Tm^3+^ UCNPs. The 976 nm laser excitation intensity was 3.76 × 10^5^ W cm^–2^, and the integration time for the spectra was 60 s. (d) Emission spectra acquired at 293 and 398 K from the same tandem UCNP pair shown in panel (b). The wavelength bounds used to calculate the temperature-dependent luminescence intensity ratios are indicated for both compositions. The excitation intensity and integration time were the same as in panel (c).

## Results and Discussion

We selected NaYF_4_:Yb^3+^,Er^3+^ and NaYF_4_:Yb^3+^,Tm^3+^ as candidate UCNP compositions for subdiffraction temperature measurements via spectral orthogonality since thermometry based on the green emission of Er^3+^ is well-established^25^ and thermometry using the blue emission of Tm^3+^ has also been reported,^26–28^ both under 976–980 nm excitation. We prepared samples consisting of hexagonally faceted NaYF_4_:Yb^3+^,Er^3+^ UCNPs (∼65 nm in diameter) and NaYF_4_:Yb^3+^,Tm^3+^ UCNPs (∼50 nm in diameter) sequentially spin coated onto a silicon substrate with fiducial markers. Figure S1 in the Supporting Information shows transmission electron microscope (TEM) images of each UCNP composition, which were used to determine the average dimensions of each composition (Figure S2), and Figure S3 shows histograms used to identify the characteristic emission intensity for single UCNPs of each composition. As indicated in Figure 1a, we define a tandem UCNP pair as one NaYF_4_:Yb^3+^,Er^3+^ and one NaYF_4_:Yb^3+^,Tm^3+^ UCNP that are located sufficiently close to one another such that both fall within the excitation laser spot and their temperature-dependent emission spectra can thus be acquired simultaneously, yet are far enough from other nearby UCNPs such that only the tandem pair will be excited. Figure 1b shows a scanning electron microscope (SEM) image of a tandem UCNP pair. The two UCNP compositions are indistinguishable under SEM imaging but are readily distinguished optically: by imaging the emitted luminescence through either a 542 ± 10 nm bandpass filter corresponding to the green Er^3+^ emission or a 475 ± 12.5 nm bandpass filter corresponding to the blue Tm^3+^ emission, we can identify pairs consisting of one UCNP of each composition and differentiate the two UCNPs from one another.

Over the 440 to 570 nm wavelength range considered in this work, NaYF_4_:Yb^3+^,Er^3+^ UCNPs predominantly emit at green wavelengths, while NaYF_4_:Yb^3+^,Tm^3+^ UCNPs predominantly emit at blue wavelengths. However, the high laser intensities required for single-particle measurements^29^ (typically ∼10^4^–10^6^ W/cm^2^, with excitation intensities of ∼(3–4) × 10^5^ W/cm^2^ used for all measurements in this work) can excite transitions involving higher-energy excited states,^30^ resulting in additional spectral peaks that could cause interference between Er^3+^ and Tm^3+^ emission. Figure 1c shows a room temperature emission spectrum acquired from the same tandem UCNP pair shown in Figure 1b, along with room temperature spectra acquired separately from isolated individual UCNPs of each composition. The emission spectra from the tandem pair and the individual UCNPs are largely in strong agreement, indicating negligible spectral crosstalk, with the exception of the ∼500–520 nm region where emission originating from the ^1^D_2_ to ^3^H_5_ transition of Tm^3+^ partially interferes with emission originating from the ^2^H_11/2_ to ^4^I_15/2_ transition of Er^3+^. As detailed below, we exclude this wavelength range in our analysis of the temperature-dependent signals to avoid confounding effects resulting from this modest spectral overlap.

Figure 1d shows emission spectra recorded at 293 and 398 K from the tandem UCNP pair shown in Figure 1b. To measure temperature using NaYF_4_:Yb^3+^,Er^3+^ UCNPs, we rely on the transitions from the closely spaced, thermally coupled ^2^H_11/2_ and ^4^S_3/2_ energy levels, whose populations are governed by Boltzmann statistics, to the ^4^I_15/2_ ground state.^31^ The resulting temperature-dependent shift in the relative emission intensity originating from these two transitions can be described by a luminescence intensity ratio defined as $r_{{\mathrm{Er}}^{3+}}≡\frac{\int_{\lambda_{1}}^{\lambda_{2}}I\left(\lambda \right)d\lambda }{\int_{\lambda_{2}}^{\lambda_{3}}I\left(\lambda \right)d\lambda }$ , where *I*(λ) is the emission spectrum. This ratiometric thermometry signal is very well-established including at the single-UCNP level.^16,25^ The wavelength bounds λ_1_, λ_2_, and λ_3_ used to calculate *r*_Er^3+^_ are also indicated in Figure 1d. Here, we select a value of λ_1_ = 520 nm that is slightly higher than typical to exclude the influence of emission originating from the ^1^D_2_ to ^3^H_5_ transition of Tm^3+^, which we later show does not impede successful use of the ratiometric thermometry signal. If we instead use a more typical value of λ_1_ = 513 nm, *r*_Er^3+^_ increases only slightly for isolated NaYF_4_:Yb^3+^,Er^3+^ UCNPs but increases much more substantially for tandem UCNP pairs due to contribution of the Tm^3+^ emission resulting from the spectral crosstalk (Figure S4). More importantly, allowing spectral crosstalk would in fact eliminate the ability to measure multiple temperature points in a subdiffraction region since *r*_Er^3+^_ would no longer report the temperature solely at the location corresponding to the NaYF_4_:Yb^3+^,Er^3+^ UCNP, but would instead represent a weighted spatial average of the temperatures at the two locations corresponding to both UCNPs in the tandem pair.

While blue Tm^3+^ emission has been studied less extensively for thermometry than green Er^3+^ emission, several ratiometric approaches have been reported. For example, Wang et al.^26^ and Burikov et al.^27^ both reported thermometry based on the ratio of the ∼450 nm emission resulting from the ^1^D_2_ to ^3^F_4_ transition relative to the ∼475–480 nm emission resulting from the ^1^G_4_ to ^3^H_6_ transition. Meanwhile, Pereira et al.^28^ demonstrated an approach based on the intensity ratio of two sub-bands of this latter emission peak that originate from different transitions involving thermally coupled Stark sublevels of the ^1^G_4_ and ^3^H_6_ manifolds, which is the approach we adopt here. Although intensity ratios involving red and near-infrared Tm^3+^ upconversion emission peaks have also employed for thermometry,^26,32^ here we focus solely on blue Tm^3+^ emission to maintain spectral proximity to the green Er^3+^ emission and avoid challenges associated with chromatic aberrations. These prior studies have all demonstrated thermometry only at the ensemble level, whereas here we report single-UCNP measurements. We define the temperature-dependent luminescence intensity ratio as $r_{{\mathrm{Tm}}^{3+}}≡\frac{\int_{\lambda_{1}}^{\lambda_{2}}I\left(\lambda \right)d\lambda }{\int_{\lambda_{3}}^{\lambda_{4}}I\left(\lambda \right)d\lambda }$ . The wavelength bounds λ_1_, λ_2_, λ_3_, and λ_4_ used to calculate *r*_Tm^3+^_ are indicated in Figure 1d. At elevated temperatures, we find that the integrated emission intensity between λ_3_ and λ_4_ decreases relative to the integrated emission between λ_1_ and λ_2_, thereby increasing *r*_Tm^3+^_, consistent with the behavior observed by Pereira et al.^28^ We also note that a general advantage of ratiometric approaches is their robustness against changes in the absolute emission intensity induced by factors including minor fluctuations in the excitation laser intensity or changes in optical alignment.

Next, we assessed the variability of the temperature-dependent emission among different isolated individual UCNPs of each composition, among different tandem UCNP pairs, and between isolated individual UCNPs and the corresponding emission from tandem pairs. We recorded the temperature-dependent responses of tandem UCNP pairs and isolated individual UCNPs from room temperature (293 K) up to 398 K. The temperature-dependent ratios we calculate for three individual NaYF_4_:Yb^3+^,Er^3+^ UCNPs and for the Er^3+^ emission from four different tandem UCNP pairs are all in good agreement (Figure 2a). There is likewise good agreement among the ratiometric thermometry signals obtained for three individual NaYF_4_:Yb^3+^,Tm^3+^ UCNPs and the Tm^3+^ emission recorded from four tandem UCNP pairs (Figure 2b). Both the absolute values and the percent increase we record for *r*_Tm^3+^_ over this temperature range are comparable to those reported by Pereira et al.^28^ for ensemble measurements, further supporting the viability of extending this approach to the single-UCNP level.

**Figure 2:**
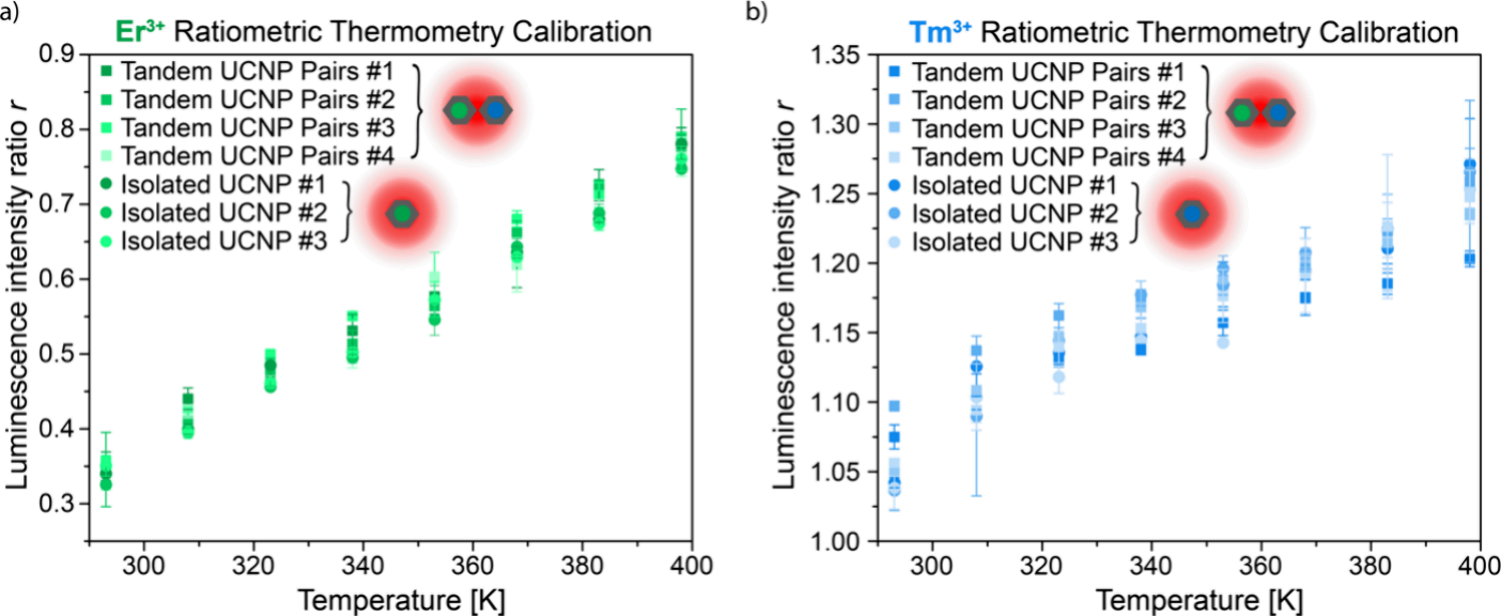
(a) Luminescence intensity ratio versus temperature calibrations for three individual NaYF_4_:Yb^3+^,Er^3+^ UCNPs and the Er^3+^ emission from four tandem UCNP pairs. Error bars represent the standard deviation of two consecutive measurements. The excitation intensity was 3.76 × 10^5^ W cm^–2^, and the integration time for all spectra was 60 s. (b) Luminescence intensity ratio versus temperature calibrations for three individual NaYF_4_:Yb^3+^,Tm^3+^ UCNPs and the Tm^3+^ emission from four tandem UCNP pairs. The excitation intensity and integration time were the same as in panel (a), and error bars again represent the standard deviation of two consecutive measurements.

Demonstrating the ability of a tandem UCNP pair to access subdiffraction temperature information requires a structure capable of generating a steep temperature gradient. To generate such a structure, we formed isolated silver (Ag) nanodisks by annealing thin Ag films deposited onto borosilicate glass coverslips. We selected Ag since it is commonly used to create nanostructures with visible wavelength plasmonic resonances. By incorporating an additional 633 nm laser into our microscopy system that serves to heat the nanodisks (Figure S5), we can generate and subsequently probe the sharp temperature gradient that forms along the glass substrate near the edge of an isolated nanodisk,^33^ as illustrated schematically in Figure 3a. The nanodisks are spaced several microns apart from one another on average, such that we can easily heat only a single nanodisk at a time. The nanodisks are ∼200 nm in diameter and ∼30 nm in thickness (Figure 3b and Figure S6).

**Figure 3:**
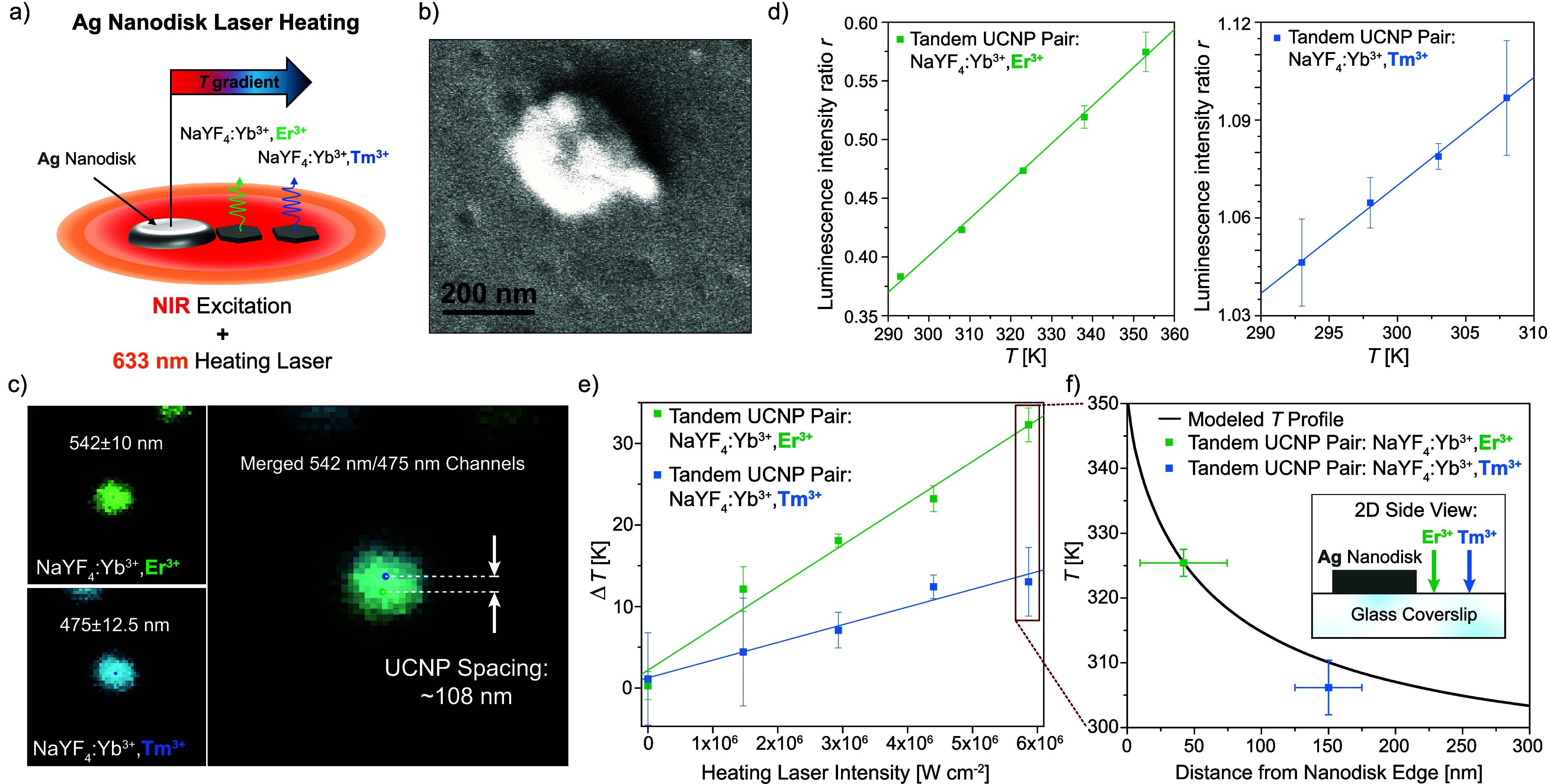
(a) Schematic depicting the use of a tandem UCNP pair to sample the steep temperature (*T*) gradient formed along a glass substrate near the edge of an isolated, laser-heated silver (Ag) nanodisk. (b) Representative SEM image of a nanodisk after depositing both NaYF_4_:Yb^3+^,Er^3+^ and NaYF_4_:Yb^3+^,Tm^3+^ UCNPs, which can be observed on and near the nanodisk. (c) Images of the emitted luminescence from a tandem UCNP pair located on the glass near the edge of an Ag nanodisk (distinct from the nanodisk shown in panel (b)). The tandem UCNP pair consists of a NaYF_4_:Yb^3+^,Er^3+^ UCNP and a NaYF_4_:Yb^3+^,Tm^3+^ UCNP spaced ∼108 nm apart from one another, with the NaYF_4_:Yb^3+^,Er^3+^ and UCNP NaYF_4_:Yb^3+^,Tm^3+^ UCNPs located ∼42 nm and ∼150 nm from the nanodisk edge, respectively. (d) Luminescence intensity ratio versus temperature calibrations for the same tandem UCNP pair imaged in panel (c) over the relevant temperature ranges for subsequent laser heating measurements. Error bars represent the standard deviation of two consecutive measurements. The excitation intensity was 3.18 × 10^5^ W cm^–2^, and the integration time for all spectra was 60 s. (e) Temperature rises measured by the tandem UCNP pair from panels (c, d) as a function of the 633 nm heating laser intensity. The temperature rise was calculated by converting the measured ratio values to temperatures and subtracting room temperature (293 K) from two consecutive measurements. Vertical error bars represent the standard deviation of the two resulting temperature rise values. The 976 nm laser intensity and integration time were the same as in panel (d). (f) Modeled temperature profile together with the highest measured temperature values, which correspond to the temperature rises recorded at the maximum 633 nm heating laser intensity shown in panel (e). Horizontal error bars represent the UCNP radii, which we take as a conservative upper bound on the UCNP position uncertainty.

We again sequentially spin coated NaYF_4_:Yb^3+^,Er^3+^ and NaYF_4_:Yb^3+^,Tm^3+^ UCNPs onto samples containing nanodisks. Figure 3b shows a representative SEM image of a nanodisk after UCNP deposition, with several UCNPs landing on or near the nanodisk in this case. However, the specific nanodisk we selected for our laser heating measurements has only one NaYF_4_:Yb^3+^,Er^3+^ and one NaYF_4_:Yb^3+^,Tm^3+^ UCNP located within the vicinity of the nanodisk, both on the adjacent glass region, as depicted in Figure 3a. The lefthand portion of Figure 3c shows images of this selected tandem UCNP pair obtained by raster scanning the same region of the sample and recording the emitted luminescence through both 542 ± 10 nm and 475 ± 12.5 nm bandpass filters. The emission spots corresponding to the particular NaYF_4_:Yb^3+^,Er^3+^ and NaYF_4_:Yb^3+^,Tm^3+^ UCNPs of interest are located at the center of each image, while portions of emission spots originating from other UCNPs can also be observed at the upper edges of both images. The righthand portion of Figure 3c merges these two images. While the diffraction limited UCNP emission spots overlap nearly completely, by locating the centroid of each spot, we can determine that the center-to-center distance between the two UCNPs is ∼108 nm. The diameters of the UCNPs determine both the area over which the temperature measured by each composition is spatially averaged and the minimum center-to-center spacing, which together determine best achievable spatial resolution of the technique. Figure S7 shows that the full width at half-maximum is similar for emission spots from both compositions at the excitation intensity of 3.18 × 10^5^ W cm^–2^ applied here. By superimposing the known centroid locations on top of a widefield image of the nanodisk (Figure S8), we also determine that the NaYF_4_:Yb^3+^,Er^3+^ is located ∼42 nm away from the edge of the nanodisk and the NaYF_4_:Yb^3+^,Tm^3+^ is thus located ∼150 nm from the edge.

Using the temperature-dependent responses recorded for each UCNP composition shown previously in Figure 2a,b, we first determined the approximate maximum temperature experienced by each UCNP at our peak 633 nm heating laser intensity. As expected, the NaYF_4_:Yb^3+^,Er^3+^ UCNP reaches a higher maximum temperature than the NaYF_4_:Yb^3+^,Tm^3+^ UCNP since it is located closer to the edge of the Ag nanodisk. We then calibrated the temperature-dependent ratios under 976 nm excitation only for the specific individual NaYF_4_:Yb^3+^,Er^3+^ and NaYF_4_:Yb^3+^,Tm^3+^ UCNPs subsequently used in our laser heating measurements (Figure 3d). Performing these calibrations only over the relevant temperature range and with the exact particles later used to probe the laser heating helps reduce the resulting temperature uncertainty. The experimental *r*_Er^3+^_ data can be fit to an Arrhenius-type relation,^16,25,31^ $r_{{\mathrm{Er}}^{3+}}=A\exp\left(-\frac{\Delta E}{k_{B}T}\right)$ , where *A* is a constant connected to the radiative transition rates from ^2^H_11/2_ and ^4^S_3/2_ to ^4^I_15/2_ and Δ*E* represents the energy difference between ^2^H_11/2_ and ^4^S_3/2_. For the *r*_Tm^3+^_ data, we follow the empirical approach of Pereira et al.^28^ and fit the data with a linear model. While we determined that applying an Arrhenius-type model would be unphysical in this case, over the narrower temperature range corresponding to the *r*_Tm^3+^_ data shown in Figure 3d, Arrhenius-type and linear fits are nearly indistinguishable (Figure S9). Fitting parameters for both data sets are given in Table S1 in the Supporting Information. The linearized sensitivities of both UCNP compositions near room temperature are similar, with *S* = d*r*/d*T* ≈ 0.3% K^–1^. Their relative sensitivities, $S_{r}=\frac{1}{r}\frac{dr}{dT}$ , near room temperature are also on the same order of magnitude (approximately 0.8% K^–1^ for NaYF_4_:Yb^3+^,Er^3+^ and 0.3% K^-1^ for NaYF_4_:Yb^3+^,Tm^3+^), comparable to previously reported values for these same compositions and for other UCNP thermometry methods.

Figure 3e shows the temperature rises measured by both UCNPs as a function of the 633 nm heating laser intensity. The NaYF_4_:Yb^3+^,Er^3+^ UCNP records a maximum temperature rise of ∼32 K, while the NaYF_4_:Yb^3+^,Tm^3+^ UCNP records a maximum temperature rise of ∼13 K. As noted previously, the higher temperature rise measured by the NaYF_4_:Yb^3+^,Er^3+^ UCNP relative to the NaYF_4_:Yb^3+^,Tm^3+^ UCNP is expected given that the NaYF_4_:Yb^3+^,Er^3+^ UCNP is located closer to the Ag nanodisk. More importantly, the ∼19 K temperature difference measured by these two UCNPs clearly demonstrates the ability of spectrally orthogonal luminescence to sample subdiffraction temperature gradients. To further verify that the measured temperatures align with the expected results, we modeled the temperature profile for the laser-heated nanodisk structure (Note S1). We first compared the temperature profile predicted by an analytical model for a uniform, disk-shaped surface heat source with a finite element model that accounts for the true thickness and thermal conductivity of the disk. The latter is necessary to accurately describe the surface temperature profile on the surface of the disk itself, although the models agree well on surface of the glass substrate, which also holds true if we consider the Gaussian shape of the heat source (Figure S10). Both the magnitude of the temperature rise and the shape of the temperature profile are largely insensitive to the Ag thermal conductivity (Figure S11) and thickness (Figure S12). Accounting for an interfacial thermal resistance between the nanodisk and substrate^33^ and the spatial averaging of the measured temperatures resulting from the finite size of the UCNP thermometers^34^ alters the modeled temperature profile on or immediately adjacent to the nanodisk but has negligible effects at the positions of our two UCNPs (Figures S13 and S14). Prior work has also established that hexagonally faceted UCNPs of similar sizes to those employed here deposited on solid substrates maintain good thermal contact with the sample surface and have negligible internal thermal resistances compared to those associated with heat dissipation into the surrounding environment;^35^ consequently, the UCNPs can accurately report the sample surface temperature. Thus, the unknown nanodisk absorption cross-section serves as the only free parameter in the modeled temperature profile shown in Figure 3f, which we adjust such that the temperature measured by the NaYF_4_:Yb^3+^,Er^3+^ UCNP matches the modeling result (see Note S1 in the Supporting Information for additional details on the modeling input parameters). The corresponding modeled temperature at the location of the NaYF_4_:Yb^3+^,Tm^3+^ UCNP agrees with the experimental value within the measurement uncertainty, providing additional validation of our measurements.

## Conclusions

In summary, we demonstrate that UCNPs with spectrally orthogonal temperature-dependent luminescence can successfully sample sharp temperature gradients at multiple points within a subdiffraction region. We establish that visible wavelength temperature-dependent emission from tandem pairs of NaYF_4_:Yb^3+^,Er^3+^ and NaYF_4_:Yb^3+^,Tm^3+^ UCNPs can be simultaneously acquired under 976 nm excitation without detrimental spectral interference. Using NaYF_4_:Yb^3+^,Er^3+^ and NaYF_4_:Yb^3+^,Tm^3+^ UCNPs located only ∼108 nm apart from one another, we resolve a ∼19 K temperature difference originating from a steep temperature gradient near a laser-heated nanodisk, a result supported by analytical and finite element modeling of the expected temperature profile. We view UCNPs as particularly advantageous for sampling subdiffraction temperature gradients via spectrally orthogonal luminescence due to the broad tunability of both their excitation and emission wavelengths via judicious selection of different lanthanide ion dopants, their narrow emission peaks that facilitate separation in the spectral domain, and the fact that numerous UCNP compositions have been studied extensively for thermometry applications. Nonetheless, this approach can in principle be extended to many other types of luminescent thermometers. While we rely here on stochastic placement of the UCNPs, future implementations of this technique could take advantage of atomic force microscope-based nanomanipulation^17^ or optical trapping^10^ approaches to deterministically place luminescent probes at desired locations. To date, AFM nanomanipulation has proven most practical for positioning individual nanoparticles on solid surfaces. However, as noted previously, the two UCNP compositions used here are indistinguishable under SEM imaging, and the same would hold true under AFM imaging. Ensuring proper placement would therefore require strategies like sequential deposition and manipulation of each UCNP species to avoid confounding different compositions.

We envision that the capabilities we demonstrate will be most beneficial in scenarios where knowledge of multiple temperature points, such as a maximum and a minimum temperature, would provide notable advantages over knowing either one of these quantities alone, and where maintaining access to the sample surface is required or desirable. One example is quantifying surface temperature rises during plasmonic and photothermal catalysis,^36–39^ where either intentional or parasitic laser heating of metal nanostructures can in some cases generate sharp temperature gradients that substantially impact the reaction,^40^ yet access to the sample surface must largely be preserved for the reacting molecules. Another potential example is direct measurement of the temperature drop across an interface^41^ using two luminescent probes placed on either side of the interface, which is relevant to fundamental nanoscale thermal transport studies^42,43^ as well as device thermal management applications. Beyond these examples, the desirable far-field optical nature of the measurements and the simpler instrumentation requirements compared to state-of-the-art nanoscale temperature mapping approaches may inspire other applications of the technique.

## Experimental Details

### Nanodisk Formation

Glass coverslips were cleaned and prepared through a multistep sonication process involving successive 10 min sonication in acetone, isopropyl alcohol, and deionized water. Samples were allowed to air-dry before undergoing a 60-s air plasma treatment in a Harrick Plasma PDC-32G Plasma Cleaner. Next, an Ag thin film was deposited onto the glass coverslip using a Denton Vacuum DESK-II DC Sputtering System and Ag sputtering target (99.99% purity), with a 15 mA current for 120 s, resulting in a nominal film thickness of 12 nm prior to annealing. The Ag nanodisks were formed by annealing the thin film in a Thermo Scientific Lindberg/Blue M Moldatherm Box Furnace for 1 h at 550 °C. To reduce charging effects, prior to SEM imaging the sample was coated with a 6 nm Pt film also using a Denton Vacuum DESK-II DC Sputtering System.

### Tandem UCNP Pair Deposition

Since the arrangement of NaYF_4_:Yb^3+^,Er^3+^ and NaYF_4_:Yb^3+^, Tm^3+^ tandem UCNP pairs relies solely on the random distribution of UCNPs across a sample, we optimized the UCNP concentrations to encourage distributions of spatially isolated single UCNPs. NaYF_4_:Yb^3+^,Er^3+^ UCNPs were diluted in cyclohexane to a concentration of 0.002 mg/mL, while NaYF_4_:Yb^3+^,Tm^3+^ UCNPs were also diluted in cyclohexane to a concentration of 0.005 mg/mL. UCNPs of both compositions were purchased from CD Bioparticles. For both UCNP compositions, 25 μL of the diluted solution was dispersed onto the sample using a spin coater (Specialty Coating Systems G3P-8).

### Optical Microscopy and Laser Heating

A custom-built confocal microscopy and spectroscopy system was used for all optical experiments. UCNP excitation was performed using a continuous wave 976 nm fiber-coupled diode laser (BL976-PAG500, Thorlabs) along with a cleanup bandpass filter (LD01-975/10-25, Semrock). A 100× dry air objective lens with a numerical aperture of 0.8 (Nikon) was used to focus the laser beam onto the sample. A 775 nm short pass dichroic mirror (zt775sp-2p-uf3, Chroma) both reflected the laser beam toward the sample and transmitted the upconverted luminescence to the detection equipment. For the laser heating experiment and temperature calibrations performed on the selected tandem UCNP pair, a 605 nm short pass dichroic mirror (DMSP605R, Thorlabs) was instead used to direct both the 633 nm HeNe heating laser (HNL210L, Thorlabs) and the 976 nm excitation laser to the sample surface while still transmitting the upconverted luminescence from both UCNP compositions. The UCNP luminescence was collected by raster scanning the sample mounted in a thermal stage (custom version of HCS321Gi, Instec) atop a piezo-controlled nanopositioning stage (Mad City Labs Nano-T115). The luminescence then passed through a short pass filter (BSP01-785R-25, Semrock) to remove any residual excitation light, and an additional 633 nm notch filter (ZET633TopNotch, Chroma) was added to remove any remaining heating laser light. After passing through a 100 μm confocal pinhole, a rotating filter wheel containing bandpass filters centered around the green Er^3+^ emission (FF01-542/20-25, Semrock) and blue Tm^3+^ emission (#87-788, Edmund Optics) controlled the wavelength range of emission that was focused onto an avalanche photodiode (Micro Photon Devices PDM Series). A spectrometer (Andor Kymera 193i spectrograph with an iDus 420 CCD camera) was used to record luminescence spectra.

## Supplementary Material



## Notes

TEM images of the UCNPs and histograms of their size distributions, histograms used to determine the characteristic single-UCNP emission intensities, analysis of spectral crosstalk effects, diagram of the optical microscopy and spectroscopy system, AFM imaging of the Ag nanodisks, analysis of single-UCNP emission spot sizes, widefield Ag nanodisk imaging overlaid with UCNP emission spot centroid locations, temperature calibration models and fitting parameters, and details of the analytical and finite element modeling (PDF)
