# Supplementary material for: Sampling Sub-Diffraction Temperature Gradients with Spectrally Orthogonal Nanoparticle Luminescence
Source: ACS Photonics. 2025 Oct 23;12(11):6468–75. doi: 10.1021/acsphotonics.5c02017 (PMC12636077; doi:10.1021/acsphotonics.5c02017)
Supplement: Supplementary file 1 [file ph5c02017_si_001.pdf]

Supporting Information:

Sampling Sub-Diffraction Temperature Gradients with Spectrally Orthogonal Nanoparticle Luminescence

Benjamin Harrington[1], Qiwen Xiao[1], Junyi Lin[2], Ashley Johnson[3,4], and Andrea D. Pickel[1,2,3,*]

[1] Materials Science Program, University of Rochester, Rochester, NY 14627

[2] Department of Mechanical Engineering, University of Rochester, Rochester, NY 14627

[3] The Institute of Optics, University of Rochester, Rochester, NY 14627

[4] Department of Physics, Texas State University, San Marcos, TX 78666

*Corresponding author. Email: apickel@ur.rochester.edu

Contents: Pages S1 – S15, Figures S1 – S14, and Table S1

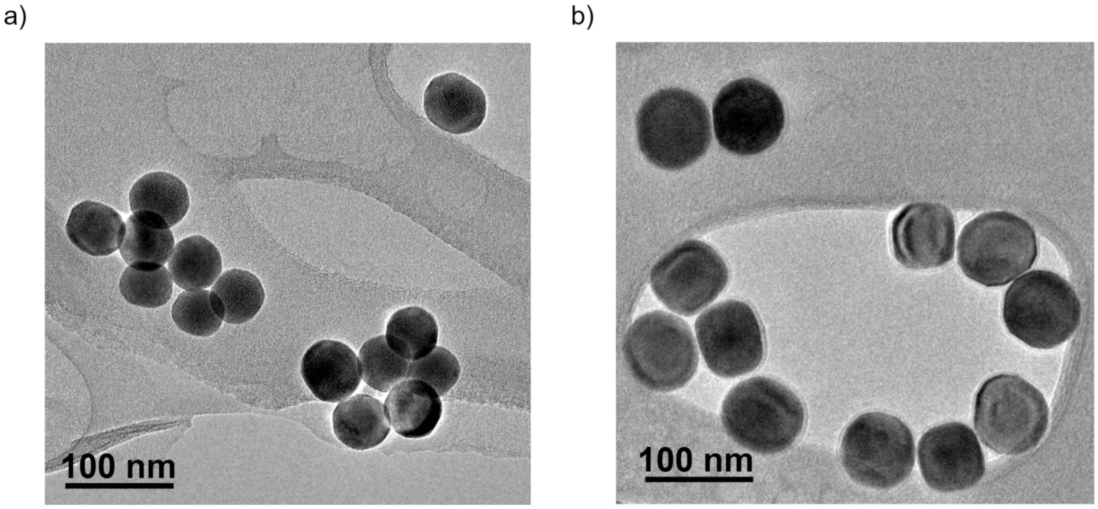

Figure S1. Representative transmission electron microscope (TEM) images of (a) $NaYF_4$:Yb^{3+},Tm^{3+} and (b) $NaYF_4$:Yb^{3+},Er^{3+} UCNPs distributed onto a TEM grid.

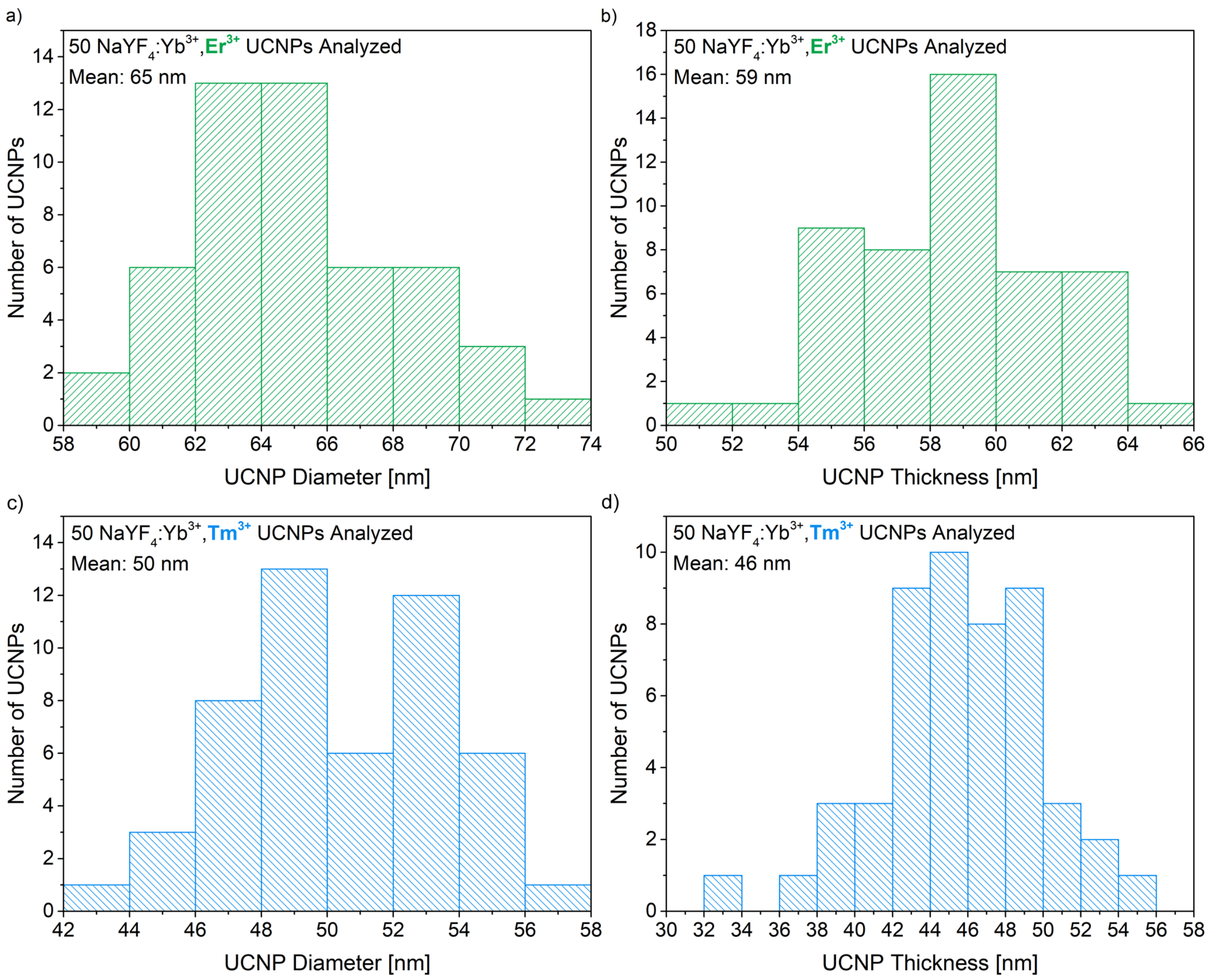

Figure S2. Size distributions analyzed from the TEM images in Figure S1 for $NaYF_4$: Yb^{3+},Er^{3+} and $NaYF_4$: Yb^{3+},Tm^{3+} UCNPs (a), (c) oriented with their hexagonal facets facing up, where the diameter is the distance across the hexagonal facet, and (b), (d) oriented on their sides, where the thickness is the distance between the two hexagonal facets.

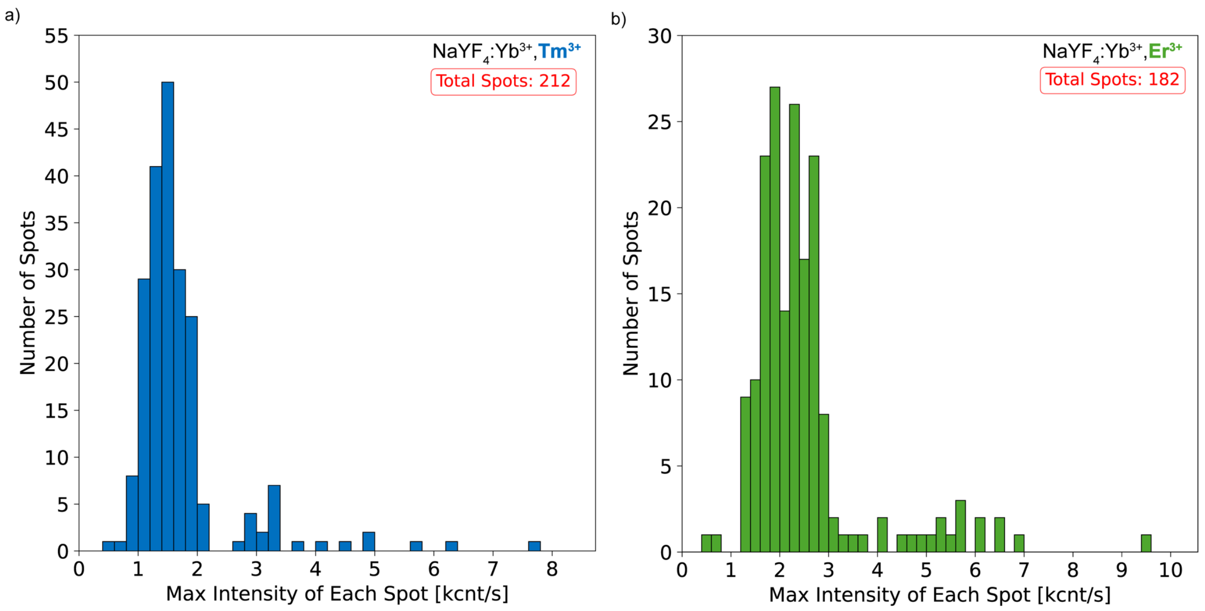

Figure S3. Histograms obtained by analyzing the peak intensities of (a) 212 emission spots for a sample containing only $NaYF_4:Yb^{3+},Tm^{3+}$ UCNPs and (b) 182 emission spots for a sample containing only $NaYF_4:Yb^{3+},Er^{3+}$ UCNPs, both dispersed on glass coverslips. The largest peaks for the two histograms, occurring between ~1500 – 2000 counts per second, indicate that both samples contain primarily well-isolated single UCNPs.

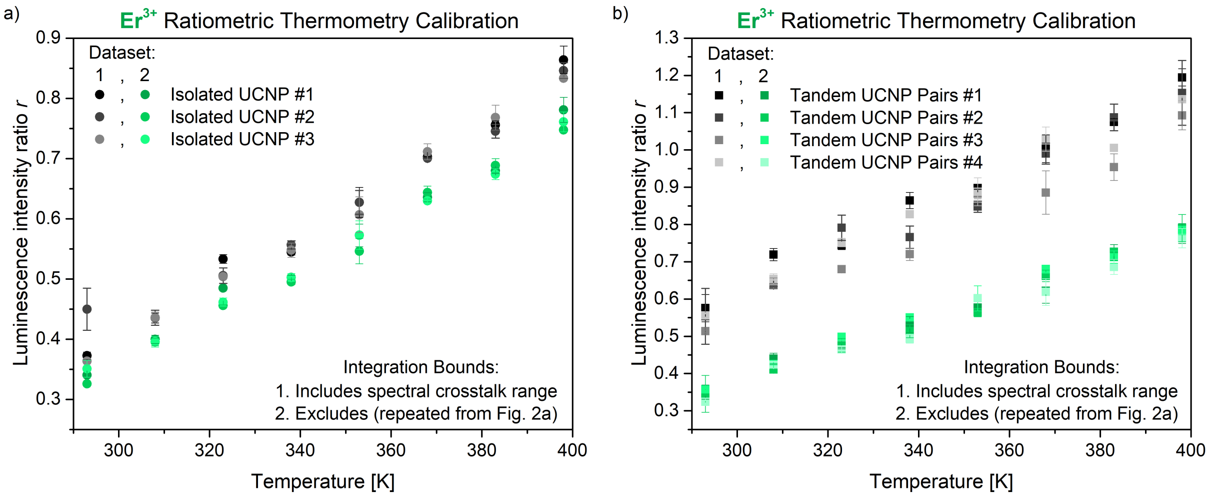

Figure S4. To characterize the effects of the spectral crosstalk resulting from overlapping Tm^{3+} and Er^{3+} emission within the ~500-520 nm wavelength range, we compare values for $r_{Er^{3+}}$ calculated using the same wavelength bounds as in main text Figure 2a (green data points), which excludes the crosstalk region, to values calculated using a more typical lower bound of λ_1 = 513 nm (black data points). (a) Reducing λ_1 results in only a modest increase in the calculated $r_{Er^{3+}}$values for isolated NaYF4:Yb^{3+},Er^{3+} UCNPs. Here, the increase originates solely from the inclusion of additional Er^{3+} emission. (b) Meanwhile, for tandem UCNP pairs, reducing λ_1 increases $r_{Er^{3+}}$ much more substantially due to the additional contribution from Tm^{3+} emission within the crosstalk region. Consequently, we exclude this wavelength region to avoid parasitic spatial averaging of the temperatures measured by the two different UCNPs that make up a tandem pair.

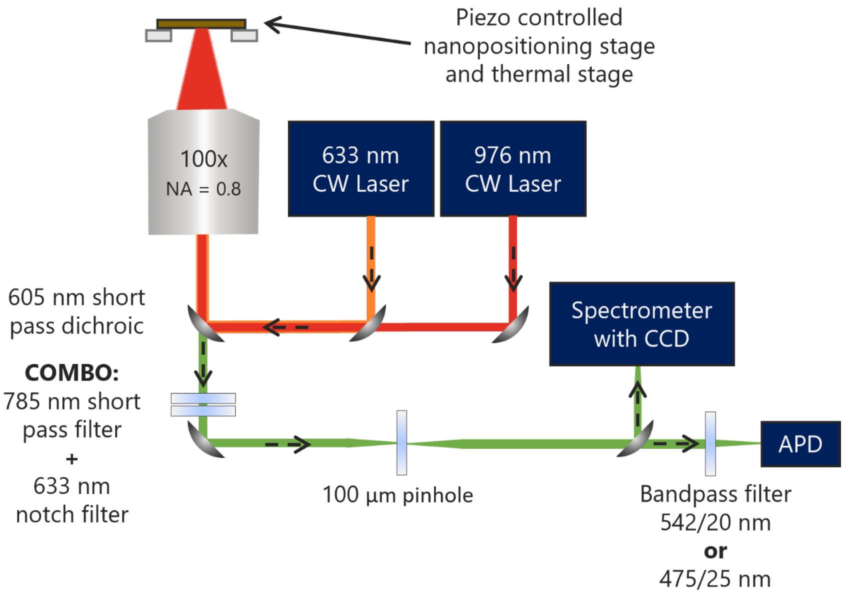

Figure S5. Custom-built microscopy and spectroscopy set-up combining a 976 nm excitation laser that excites the UCNPs and a 633 nm laser used to heat the Ag nanodisk.

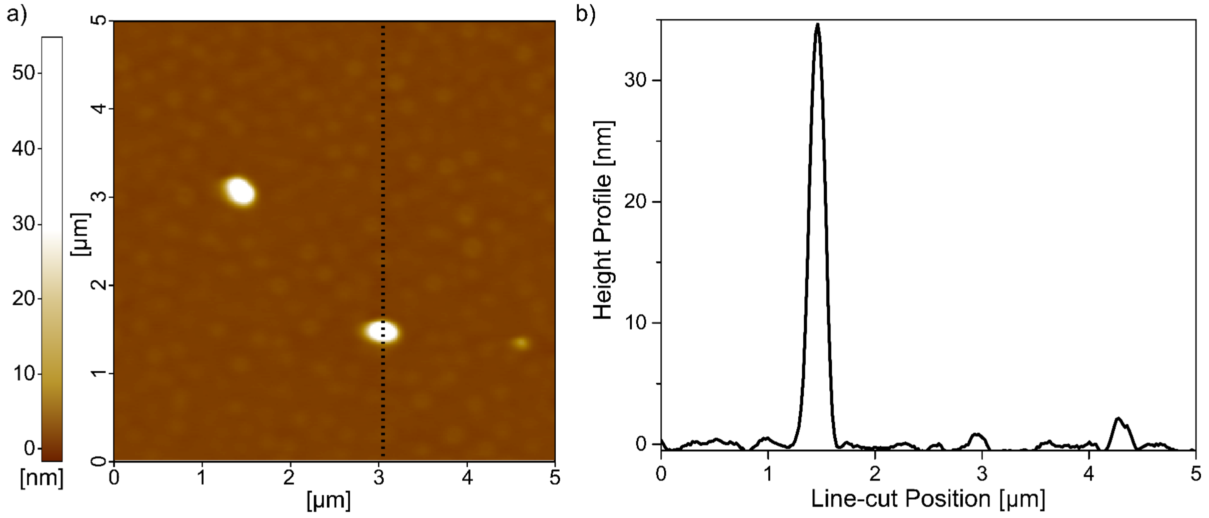

Figure S6. (a) Atomic force microscope (AFM) scan of Ag nanodisks on a glass coverslip. The nanodisks are distributed several microns apart on average. (b) Height profile along the dashed line denoted in (a), showing that the selected nanodisk is slightly over 30 nm in height.

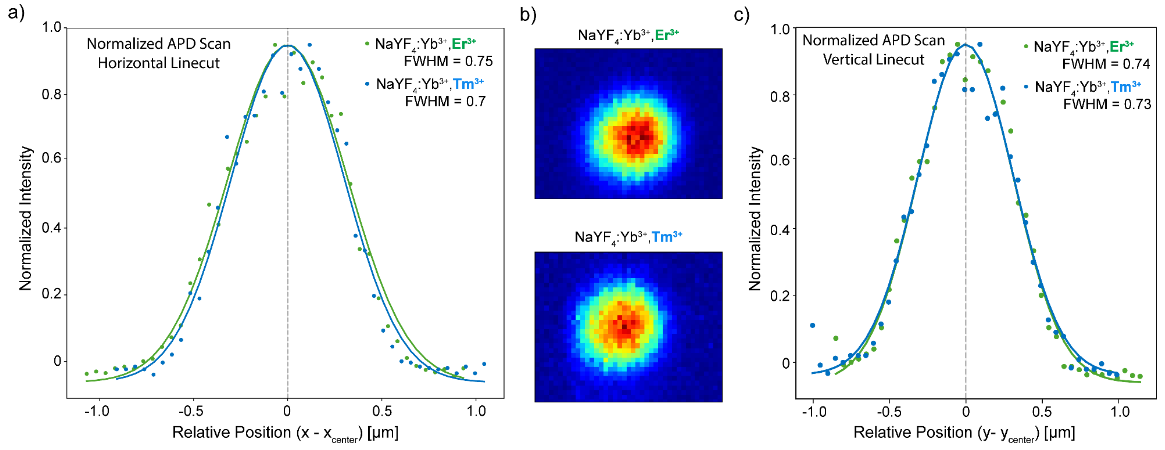

Figure S7. UCNP emission spot size for $NaYF_4$:Yb^{3+},Tm^{3+}, and $NaYF_4$:Yb^{3+},Er^{3+} UCNPs along (a) a horizontal linecut and (c) a vertical linecut. The emission intensity collected from an avalanche photodiode (APD) image of each UCNP composition is shown in (b). The full width at half maximum (FWHM) is similar for both $NaYF_4$:Yb^{3+},Tm^{3+} and $NaYF_4$:Yb^{3+},Er^{3+} UCNPs, and along both the horizontal and vertical directions. The FWHM is consistent with the diffraction limited nature of each UCNP emission spot.

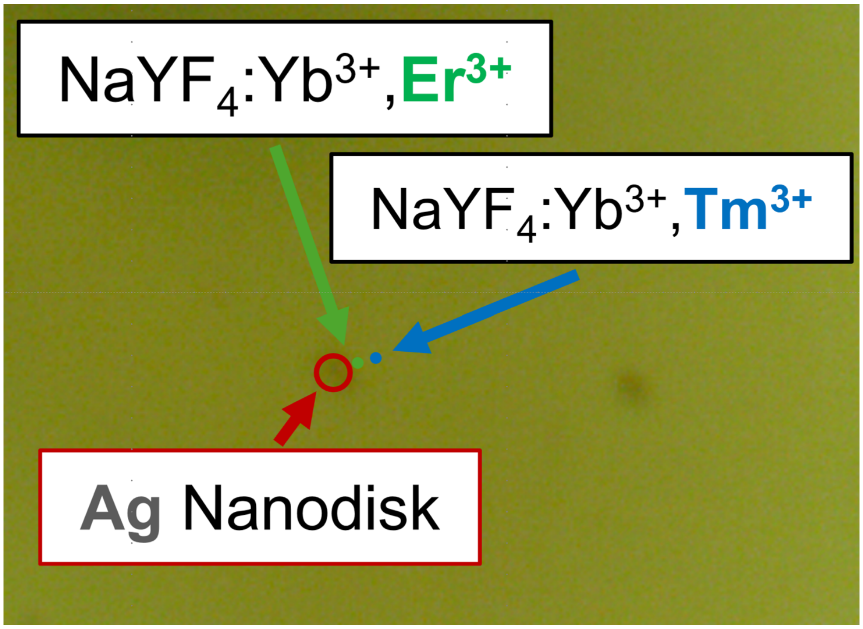

Figure S8. Composite widefield image indicating the centroid locations of emission spots from a tandem pair of $NaYF_4$:Yb^{3+},Er^{3+} (green dot) and $NaYF_4$:Yb^{3+},Tm^{3+} (blue dot) UCNPs relative to an Ag nanodisk (bounded by the red outline). The UCNP centroid locations were determined by first mapping their emission intensities with an APD while raster scanning the sample using a nanopositioning stage (see main text Figure 3c), since the UCNPs are not resolvable in our widefield imaging mode due to their small size and the diffraction limited nature of the imaging system.

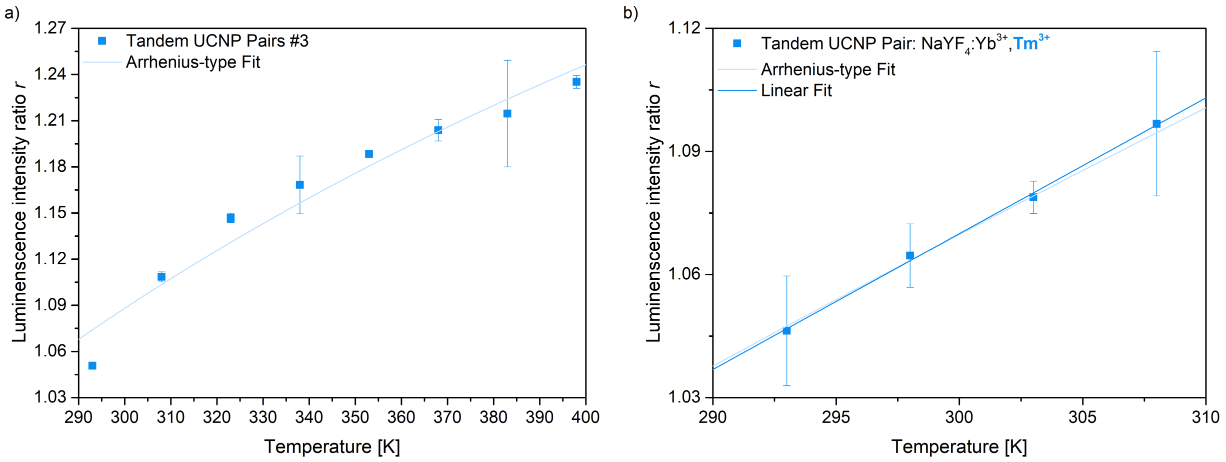

Figure S9. (a) Applying an Arrhenius-type model to the experimental $r_{Tm^{3+}}$ data results in a poor fit over the full temperature range used for the calibrations shown in main text Figure 2. Because the fitting parameters associated with this type of model have physically meaningful interpretations related to the Tm^{3+} energy levels and transitions, a poor Arrhenius-type fit would lead to an unphysical interpretation. (b) While we therefore selected linear fitting as a more appropriate option, Arrhenius-type and linear fits are nearly indistinguishable over the narrower temperature range where the fitting was performed in main text Figure 3d.

**Table S1.** Selected Tandem UCNP Pair Ratiometric Thermometry Calibration Fitting Parameters		
**Boltzmann Fitting Parameters:**	**A**	**ΔE [meV]**
$NaYF_4$:Yb^{3+},Er^{3+}	4.22	61
**Linear Fitting Parameters:**	**Slope [K^{-1}]**	**Intercept**
$NaYF_4$:Yb^{3+},Tm^{3+}	0.00331	0.07639

Note S1: Modeling the Temperature Profile

To contextualize the temperature values experimentally measured by the tandem UCNP pair in main text Figure 3, analytical and finite element models were developed. For the analytical model, we treat the sample as a semi-infinite solid and solve the steady state heat conduction equation in cylindrical coordinates,

$$\frac{1}{r}\frac{\partial}{\partial r}\left(k_{sub} r \frac{\partial \theta(r,z)}{dr}\right) + \frac{\partial}{\partial z}\left(k_{sub}\frac{\partial \theta(r,z)}{\partial z}\right) = 0, \tag{S1}$$

where k_{sub} is the thermal conductivity of the glass substrate (taken to be constant) and $\theta(r,z) = T(r,z) - T_\infty$ is the temperature rise with respect to the far-field ambient temperature T_∞. One boundary condition describes the constant heat flux Q applied to a circular disk-shaped region of radius R on the top surface,

$$-k_{sub}\frac{\partial \theta}{\partial z}(r, z = 0) = Q, 0 \le r \le R, \tag{S2}$$

with zero heat flux applied on the top surface for $r > R$, while the other boundary conditions specify that far from this heat source the temperature approaches the ambient value, T_∞. This type of axisymmetric problem can be solved using Hankel transforms. Taking the Hankel transform of Eq. S1 yields

$$\frac{\partial^2 \theta'(s,z)}{\partial z^2} - s^2\theta'(s,z) = 0, \tag{S3}$$

where s is the Hankel transform variable and $\theta'(s,z)$ is the Hankel transform of the temperature rise. Solving Eq. S3 gives the following general solution,

$$\theta'(s,z) = A(s)e^{-sz} + B(s)e^{sz}. \tag{S4}$$

To fulfill the condition that the temperature rise must remain finite as $z \to \infty$, we set $B(s) = 0$. Taking the Hankel transform of Eq. S2 yields

$$-k_{sub}\frac{\partial \theta'}{\partial z}(s, z = 0) = \frac{QRJ_1(sR)}{s}, \tag{S5}$$

where J_1 is the first-order Bessel function of the first kind. Combining Eq. S5 with the fact that $\frac{\partial \theta'}{\partial z}(s, z = 0) = -sA(s)$ as can be determined from Eq. S4, we find that $A(s) = \frac{QR}{ks^2}J_1(sR)$ and we thus obtain the following solution for $\theta'(s,z)$:

$$\theta'(s,z) = \frac{QR}{ks^2}J_1(sR)e^{-sz}. \tag{S6}$$

Finally, taking the inverse Hankel transform of Eq. S6 yields the following result for the temperature rise $\theta(r,z)$,

$$\theta(r,z) = \frac{QR}{k}\int_0^{\infty}\frac{J_0(sr)J_1(sR)}{s}e^{-sz}ds, \quad \text{(S7)}$$

where J_0 is the zeroth-order Bessel function of the first kind. The surface temperature, which is the quantity corresponding to our experimental measurements, can be obtained by taking $T(r,z) = \theta(r,z) + T_\infty$ and setting $z = 0$. The integral in Eq. S7 can be evaluated numerically, and in practice we find that the upper bound can be set to $100/R$ for the calculations performed in this work without loss of accuracy.

Finite element analysis was carried out in COMSOL using a 2D representation of the experimental geometry due to the radial symmetry about the center of the nanodisk. The nanodisk was given a radius R of 100 nm and placed atop a semi-infinite glass substrate with a thermal conductivity k_{sub} of 1 W m^{-1} K^{-1}. A heat flux was applied to the top surface of the nanodisk, the bottom surface of the glass was fixed at T_∞ = 293 K, and all other surfaces were treated as adiabatic. The nanodisk thickness and silver (Ag) thermal conductivity were set to 30 nm and 200 W m^{-1} K^{-1}, respectively, unless otherwise specified.

Figure S10 compares the temperature profiles predicted (i) by the analytical model for a uniform, disk-shaped surface heat source of radius R applied to the top surface of the semi-infinite substrate and (ii) by the finite element model for both a uniform heat flux and a Gaussian heat flux applied to the top surface of the nanodisk. For the analytical model, we consider the temperature rise along the top surface of the glass substrate. For the finite element model, we consider the temperature rise along the top surface of the Ag nanodisk for $r \leq R$ and along the top surface of the glass substrate for $r > R$, corresponding to the experimentally accessible surface temperatures in our measurements. The analytical solution overpredicts the peak temperature rise and cannot account for spatial uniformity of the temperature rise along the nanodisk, both of which result from the much higher thermal conductivity of the Ag nanodisk relative to the glass substrate. However, the modeled temperature profiles are all in excellent agreement over the region where the UCNP temperature measurements shown in main text Figure 3 were performed (≥ 142 nm from the center of the nanodisk).

The thermal conductivity of thin Ag films will be reduced from the corresponding bulk Ag value due to effects such as boundary and defect scattering. Based on reported literature values for Ag thin film thermal conductivities[1-4], we consider a conservatively wide range of 100 – 400 W m^{-1} K^{-1}. Figure S11 shows that the finite element modeling results display negligible differences across this range of Ag thermal conductivities. While we experimentally characterize the typical nanodisk thickness through AFM measurements as shown previously in Figure S6, we also vary the nanodisk thickness between 20 and 40 nm and again find that these changes have a negligible effect on the temperature profile (Figure S12).

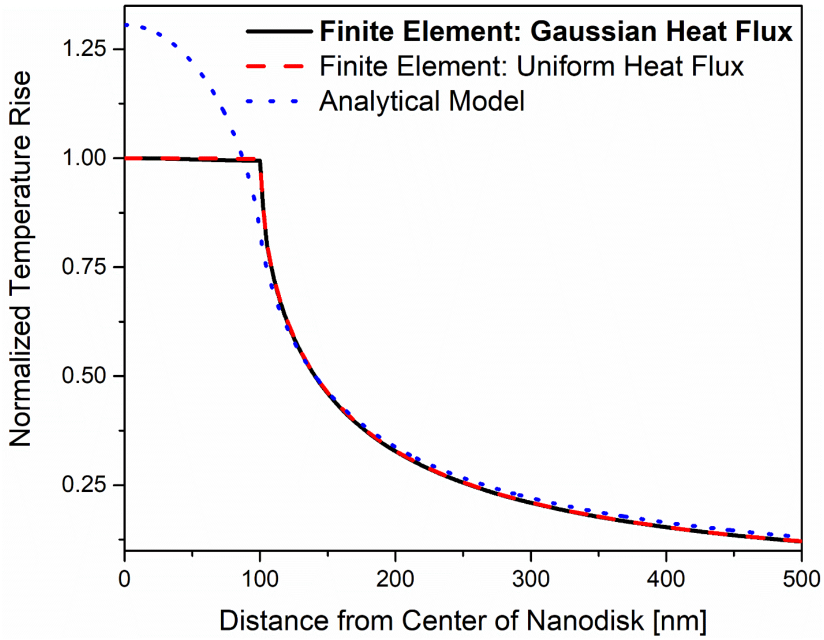

Figure S10. Analytical modeling of the surface temperature profile for a disk-shaped surface heat source on a semi-infinite substrate and finite element modeling of the surface temperature profile along the Ag nanodisk and glass substrate for both uniform and Gaussian heat fluxes applied to the nanodisk surface.

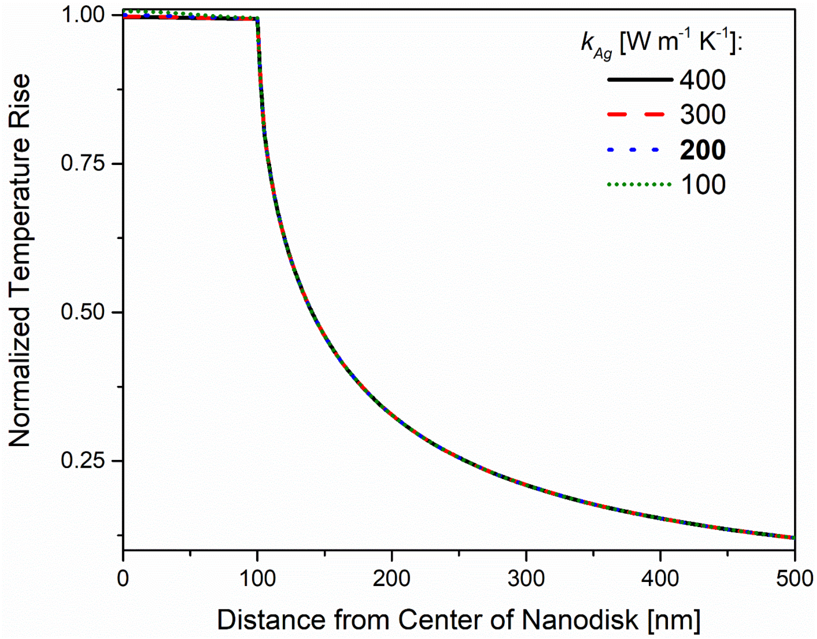

Figure S11: Finite element modeling of the surface temperature profile for varying Ag nanodisk thermal conductivities.

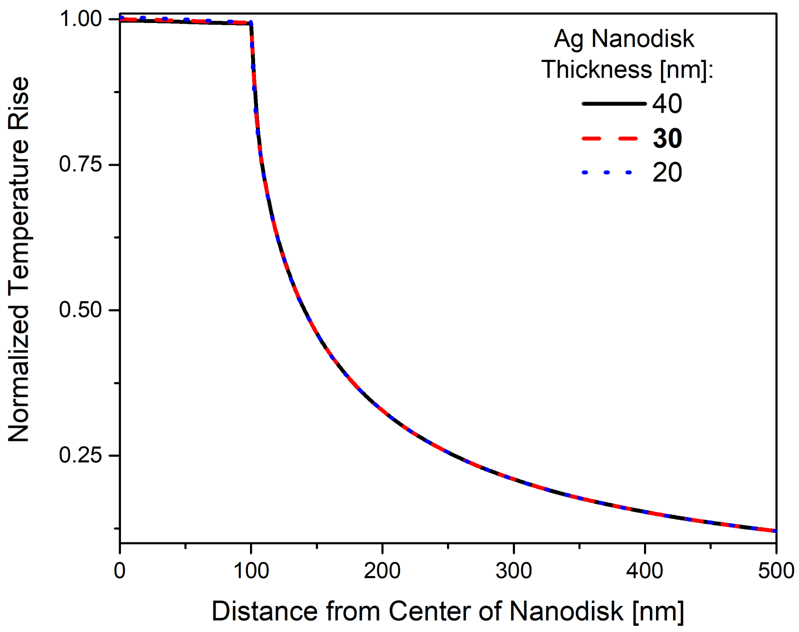

Figure S12: Finite element modeling of the surface temperature profile for varying Ag nanodisk thicknesses.

Consistent with prior work, an interfacial thermal resistance was applied between the Ag nanodisk and the glass substrate since the Kapitza length associated with the interfacial thermal resistance can be comparable to the nanodisk radius, resulting in a large temperature drop between the nanodisk and the substrate[5]. We selected a representative value of 10^{-8} m^2 K W^{-1} based on that experimentally determined by Xie and Cahill[5] for a ~174 nm diameter Au nanodisk on a quartz substrate. As expected, incorporating an interfacial thermal resistance increases the magnitude of the temperature rise along the nanodisk surface. However, in the region where the UCNP temperature measurements shown in main text Figure 3 were performed (≥ 142 nm from the center of the nanodisk), the temperature profiles agree, which would also hold true for other values of the interfacial thermal resistance.

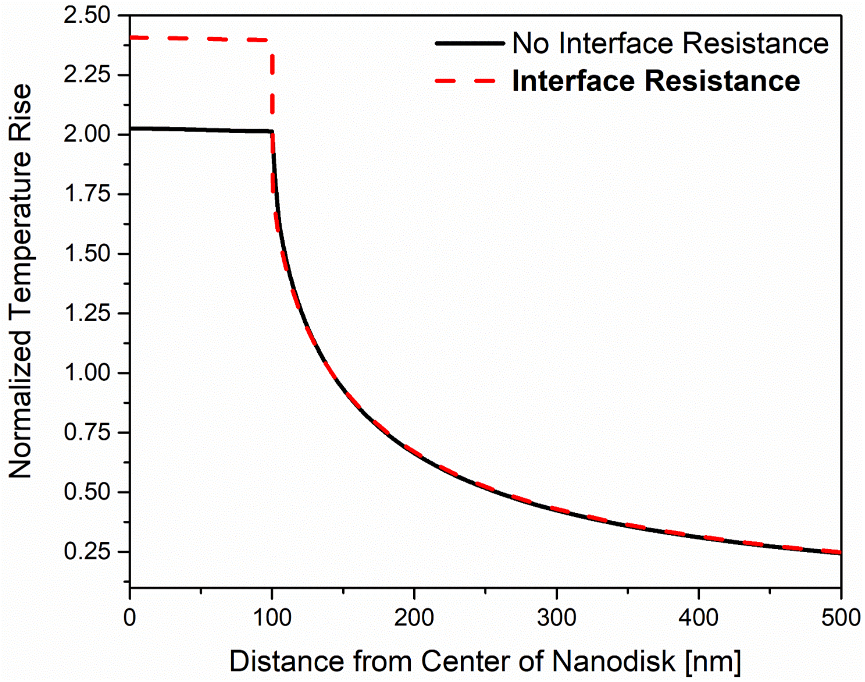

Figure S13. Comparison of finite element modeling of the surface temperature profiles with and without an interfacial thermal resistance (10^{-8} m^2 K W^{-1}) between the Ag nanodisk and the glass substrate. Here, the results are normalized by the temperature rise 142 nm from the center of the nanodisk (corresponding to location of the $NaYF_4$:Yb^{3+},Er^{3+} UCNP in main text Figure 3) to demonstrate how the interfacial thermal resistance specifically increases the temperature rise along the nanodisk.

Finally, we also consider the fact that our experimental measurements do not report the true sample temperature at a given spatial point, but rather the temperature averaged over the spatial extent of the thermometer[6]. Although the constituent UCNPs in our tandem pairs have average diameters ≤ 65 nm, the temperature profile produced by the laser-heated Ag nanodisk is very steep, with the temperature changing by tens of degrees within 200 nm from the nanodisk edge; thus, it is potentially important to consider the impact of such spatial averaging effects.

Since the activator ions that give rise to the UCNP temperature response are distributed evenly throughout the UCNP[7], the measured temperatures from the UCNPs thus correspond to a uniformly weighted average temperature over the UCNP area[6]. Figure S14 shows the results of applying such spatial averaging to the modeled temperature profiles both in the case of zero interfacial thermal resistance and an interfacial thermal resistance of 10^{-8} m^2 K W^{-1} using an averaging kernel size set to the larger of the two UCNP diameters (65 nm). The greatest deviations between the true and the spatially averaged temperature profiles arise closest to the nanodisk edge due to the sharp temperature change going from the edge of the nanodisk to the glass surface. Critically, the true and spatially averaged temperature profiles are in good agreement further away from the nanodisk edge where the measurements in main text Figure 3 were performed, with a maximum difference of 0.5%.

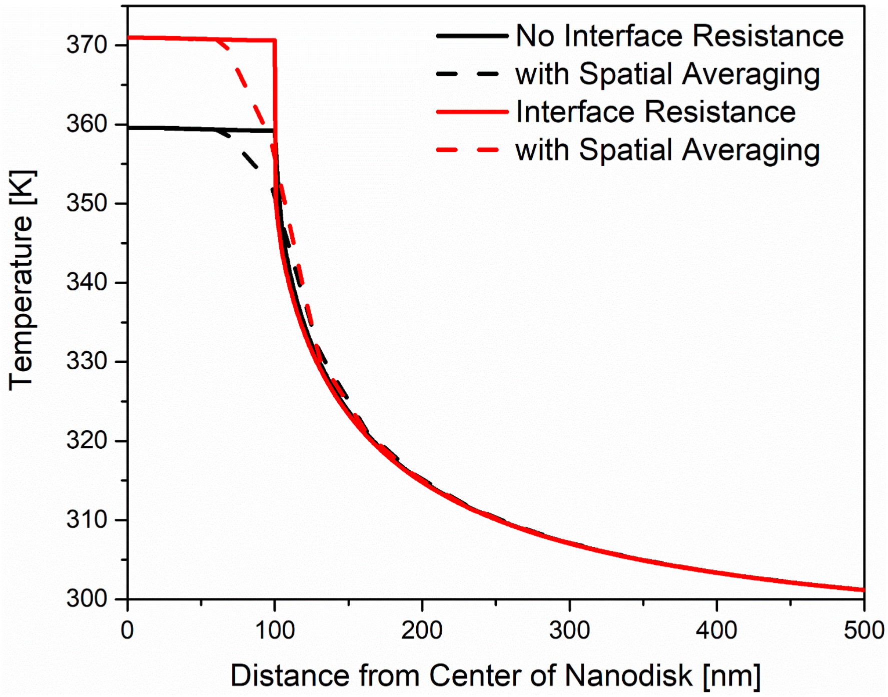

Figure S14: Comparison of the true surface temperature profiles with surface temperatures profiles accounting for the spatially averaged temperatures measured by the UCNPs, both in the case of zero interfacial thermal resistance and an interfacial thermal resistance of 10^{-8} m^2 K W^{-1} between the nanodisk and substrate.

The modeling results plotted together with the experimental UCNP data in main text figure 3f represent a finite element simulation performed with a Gaussian heat flux and an interfacial thermal resistance of 10^{-8} m^2 K W^{-1} applied between the nanodisk and glass substrate, although as detailed above, there are negligible differences in the temperature profile over the region shown in main text figure 3f for any of the model variants we consider.

Supporting Information References:

(1) Boiko, B. T.; Pugachev, A. T.; Bratsychin, V. M. Method for the determination of the thermophysical properties of evaporated thin films. *Thin Solid Films* **1973**, *17* (2), 157-161. DOI: DOI: 10.1016/0040-6090(73)90124-7.

(2) Bourgoin, J.-P.; Allogho, G.-G.; Haché, A. Thermal conduction in thin films measured by optical surface thermal lensing. *Journal of Applied Physics* **2010**, *108*, 073520. DOI: 10.1063/1.3490185.

(3) Kim, D.-m.; Nam, J.; Lee, B. J. Plasmon thermal conductivity of thin Au and Ag films. *Physical Review B* **2023**, *108*, 205418. DOI: 10.1103/PhysRevB.108.205418.

(4) Ryu, S.; Juhng, W.; Kim, Y. Effect of Microstructure on Thermal Conductivity of Cu, Ag Thin Films. *Journal of Nanoscience and Nanotechnology* **2010**, *10*, 3406-3411. DOI: 10.1166/jnn.2010.2302.

(5) Xie, X.; Cahill, D. G. Thermometry of plasmonic nanostructures by anti-Stokes electronic Raman scattering. *Applied Physics Letters* **2016**, *109*, 183104. DOI: 10.1063/1.4966289.

(6) Pickel, A. D.; Dames, C. Size and shape effects on the measured peak temperatures of nanoscale hotspots. *Journal of Applied Physics* **2020**, *128*, 045103. DOI: 10.1063/5.0012167.

(7) Gargas, D. J.; Chan, E. M.; Ostrowski, A. D.; Aloni, S.; Altoe, M. V. P.; Barnard, E. S.; Sanii, B.; Urban, J. J.; Milliron, D. J.; Cohen, B. E.; et al. Engineering bright sub-10-nm upconverting nanocrystals for single-molecule imaging. *Nature Nanotechnology* **2014**, *9*, 300-305. DOI: 10.1038/nnano.2014.29.
